# Income differences in screening, incidence, postoperative complications, and mortality of thyroid cancer in South Korea: a national population-based time trend study

**DOI:** 10.1186/s12885-020-07597-4

**Published:** 2020-11-11

**Authors:** Hee-Yeon Kang, Ikhan Kim, Yeon-Yong Kim, Jinwook Bahk, Young-Ho Khang

**Affiliations:** 1 Institute of Health Policy and Management, Seoul National University Medical Research Center, Seoul, Republic of Korea; 2 Department of Health Policy and Management, Seoul National University College of Medicine, Seoul, Republic of Korea; 3 Department of Health Policy and Management, Jeju National University School of Medicine, Jeju, Republic of Korea; 4 Big Data Steering Department, National Health Insurance Service, Wonju, Republic of Korea; 5 Department of Public Health, Keimyung University, Daegu, Republic of Korea

**Keywords:** Incidence, Income, Medical overuse, Overdiagnosis, Postoperative complication, Thyroid cancer

## Abstract

**Background:**

The incidence of thyroid cancer (TC) has increased rapidly over the past few decades in Korea. This study investigated whether the TC epidemic has been driven by overdiagnosis.

**Methods:**

We calculated the TC screening rate from mid-2008 through mid-2014, and the incidence, postoperative complication, and mortality rates of TC between 2006 and 2015, using data from the Korea Community Health Survey, the National Health Insurance Database, and the cause-of-death data of Statistics Korea. Trends in age-standardized rates of all indicators were examined, along with income gaps therein. Analyses were conducted for lung cancer and stroke as negative control outcomes.

**Results:**

The incidence rate of TC increased from 46.6 per 100,000 to 115.0 per 100,000 between 2006 and 2012, and then decreased to 63.5 per 100,000 in 2015. Despite these remarkable changes in incidence, mortality did not fluctuate during the same period. High income was associated with high rates of screening, incidence, and postoperative complications, while low income showed an association with a high mortality rate. Analyses using negative control outcomes showed that high income was associated with low rates of both incidence and mortality, which contrasted with the patterns of TC. The recent decreases in TC incidence and postoperative complications, which reflect societal concerns about the overdiagnosis of TC, were more pronounced in high-income individuals than in low-income individuals.

**Conclusions:**

The time trends in income gaps in screening, incidence, postoperative complications, and mortality of TC, as well as negative control outcomes, provided corroborating evidence of TC overdiagnosis in Korea.

**Supplementary Information:**

The online version contains supplementary material available at 10.1186/s12885-020-07597-4.

## Background

Thyroid cancer (TC) incidence has increased in many countries around the world over the last few decades [1–3]. Its incidence in South Korea (hereafter ‘Korea’) has soared to an epidemic level [4–6]. In Korea, the incidence of TC in 2015 was 7 times higher than in 1999, and the incidence peaked in 2012, with a rate 12 times higher than was observed in 1999 [7]. Increases in TC incidence rates with concomitant lack of changes in TC mortality rate have been considered as evidence for TC overdiagnosis [1–4]. Concerns about the overdiagnosis of TC have been raised in Korea. In 2010, the Korean Thyroid Association revised the management guidelines for patients with thyroid nodules and TC, including limitations on the use of fine needle aspiration cytology [8]. In 2014, the problem of the increase in TC incidence received media attention in Korea [9]. Some Korean doctors publicly pointed out the problems associated with screening for TC, arguing that it was urgent for the Korean government and medical community to restrict overdiagnosis and to take preventive measures against this issue [10]. Recently in Korea, the frequency of surgery for TC nosedived [11]. As a consequence, TC dropped from the most commonly diagnosed type of cancer to the third most commonly diagnosed type of cancer in 2015 in Korea [7]. However, a debate continues over the overdiagnosis of TC in Korea [12].

Overdiagnosis may occur more frequently in high-income strata than in low-income strata because individuals with high income are likely to use more medical services than those with low income, and they are more exposed to the risk of overutilization than low-income individuals [13, 14]. More sensitive tests tend to be carried out in high-income patients in the screening process, and such measures may lead to an earlier diagnosis, thus increasing the possibility of overdiagnosis [14–16]. Thus, discordant patterns between the relationship of income with TC incidence and the relationship of income with TC mortality could be used as the corroborating evidence for TC overdiagnosis [13].

This study is based on the hypothesis that the rates of screening, incidence, and postoperative complications of TC would be higher in high-income individuals with greater access to medical care, whereas the mortality rate would show a different pattern. In particular, recently, rising concerns about the overdiagnosis of TC in Korea have led to decreases in both the incidence of TC and surgery for TC [10], and it was hypothesized that high-income individuals would respond more quickly to social concerns. Furthermore, it was assumed that major causes of death—lung cancer (as a representative of cancers) and stroke (as a representative of cardiovascular diseases)—have relatively a low probability of overdiagnosis, and that the incidence and mortality of lung cancer and stroke by income level would therefore show a different pattern from those of TC.

## Methods

### Data

The study used data from the Korea Community Health Survey (KCHS) conducted in 2010, 2012, and 2014 by the Korea Centers for Disease Control and Prevention, data from the National Health Information Database (NHID) of National Health Insurance Service (NHIS) between 2001 and 2015, and cause-of-death statistics from Statistics Korea between 2006 and 2015. The KCHS is a nationally representative community-based cross-sectional health survey that targeted adults over 19 years of age [17]. The screening rate of TC was drawn from the KCHS items regarding TC screening. The incidence, mortality, and postoperative complication rates of TC, as well as the incidence and mortality rates of the negative control outcomes, were derived from the NHID and cause-of-death data. The NHID covers whole Korean population and includes income-based insurance premiums, demographic variables, information on the date of death, and information on healthcare utilization, thereby making it possible to analyze the incidence of diseases, medical care utilization, and mortality [18]. The study analyzed the mortality rate using individual linkage between the NHID data and cause-of-death data. Individual linkage of the raw data was conducted within the NHIS, and researchers utilized aggregate data without personal identifiers for the analysis.

This study was approved by the National Health Insurance Service of Korea (NHIS-2018-1-191) and the Seoul National University Hospital Institutional Review Board (IRB No. E-1805-047-944). Informed consent was waived by the board.

### Definitions of thyroid cancer screening, incidence, and mortality

TC examinees were defined as those who responded “yes” to an item including “thyroid cancer” among questions regarding all types of cancer screening (including national cancer screening and opportunistic screening) in the most recent 2 years from the KCHS data. All survey participants in KCHS, regardless of their baseline health status, responded to the question. To calculate the incidence rate of TC in each year, the study targeted patients who were first diagnosed with TC (ICD-10 code: C73) as a primary or secondary diagnosis every year between 2006 and 2015. The first day of treatment was considered as the date of diagnosis each year, and the study targeted patients who were admitted for TC once or more in a year or who received outpatient medical services for TC more than twice. However, patients with a history of any type of treatment for TC in the 5 years before the date of diagnosis (e.g., any history between January 1, 2001 and December 31, 2005 for an incident case on January 1, 2006) were considered to have a prior history of TC and were therefore excluded from the calculation of the incidence rate for that year. The number of cases and the age-standardized incidence rates between 2006 and 2015 derived from the NHID using the method described above were very close to those based on the Korea Central Cancer Registry (KCCR) (Supplementary Table S1). Since information on income, medical care utilization, and complications cases was not available in the KCCR, we used the NHID for this study. TC Mortality was defined as instances of people over 20 years of age with TC (ICD-10 code: C73) as the cause of death by year between 2006 and 2015.

### Definition of thyroid cancer surgery-specific complications

TC surgery-specific complications are defined as those specifically due to TC surgery, including hypoparathyroidism/hypocalcemia and vocal cord/fold paralysis [19]. General complications include postoperative fever, local complications (e.g. infection, hemorrhage, and hematoma), cardiopulmonary complications (e.g. pneumonia), and myocardial infarction, which commonly appears within 30 days after surgery [19]. The incidence of postoperative complications was calculated using patients with TC surgery-specific complications. Diagnostic and surgical procedure codes were confirmed in the healthcare utilization database of the NHID; thus, for this study, patients aged 20 or over who first underwent surgery for TC between 2006 and 2015 were included. Among those patients, postoperative complications were considered to have occurred in individuals who were hospitalized more than once or who used outpatient medical services once or more due to hypoparathyroidism (ICD-10 code: E20.9, E89.2) or vocal cord/fold paralysis (ICD-10 code: J38.0) within 31 to 365 days from the TC surgery date. However, the postoperative complication rate did not include those who used medical services due to the aforementioned complications within only 30 days after surgery, because such instances of complications were considered to have been transient symptoms in accordance with previous studies [19]. Additionally, the postoperative complication rate did not include those who had undergone any treatment for the aforementioned complications during 5 years before surgery, because such patients were considered to have a past medical history. The annual rates of TC surgery by income were also calculated. Thyroid cancer surgery included radical operations on malignant thyroid tumors, total thyroidectomy, and subtotal thyroidectomy, as well as the excision of cervical lymph nodes and neck lymphatic dissections that were performed as part of combined-modality treatment due to cervical lymph node metastasis.

### Negative control outcomes

The negative control outcomes were lung cancer and stroke since they were assumed to be less susceptible to overdiagnosis. The incidence and mortality of both lung cancer and stroke were defined similarly to those of TC. In the annual healthcare utilization database of NHID, incident cases of lung cancer were defined as individuals 20 years old or over who were first diagnosed with lung cancer (ICD-10 code: C33-C34) between 2006 and 2015. This study targeted patients who were hospitalized for lung cancer at least once or had outpatient visits twice or more for a year from the diagnosis date, as for cases of thyroid cancer. Then, patients with a past history – those who have been treated for lung cancer during the five years before the diagnosis date – were excluded. The number of cases and the age-standardized incidence rates of lung cancer from the NHID were also very close to those based on the KCCR (Supplementary Table S2). Using the annual healthcare utilization database between 2006 and 2015, incident cases of stroke were defined by identifying patients 30 years of age or over who had received their first diagnosis of stroke (ICD-10 code: I60-I64) in that year and including those were hospitalized with stroke more than once in each year. In addition, patients who had admitted for stroke the prior 5 years in each year between 2006 and 2015 were excluded because they were considered to have a past medical history. As with mortality from thyroid cancer, cases of mortality from lung cancer and stroke were defined based on linkage between the cause-of-death data from Statistics Korea and the NHID data. Those 20 years of age or older with an ICD-10 cause-of-death code of C33-C34 and those 30 years of age or older with an ICD-10 cause-of-death code of I60-I64 were defined as cases of mortality from lung cancer and stroke, respectively.

### Income

Household income from the KCHS data and national health insurance (NHI) premiums from the NHID data were used as an income index. Many previous studies in Korea have used the NHI premiums as an indicator of income [20, 21]. For the income index of TC examinees in the KCHS data, this study utilized converted values of equivalized income, in which items related to annual or monthly household income were adjusted for household size. The income index of the incidence, mortality, and postoperative complications of TC and the negative control outcomes was calculated using equivalized income adjusted for household size using NHI premium data from the NHID. In addition, each equivalized income parameter drawn from the KCHS or NHID was divided into income quintiles by year, sex, and 5-year age group [22].

### Statistical analysis

The changing patterns of the age-standardized screening, incidence, surgery, postoperative complication, and mortality rates of TC were analyzed by sex and income level between 2006 and 2015. For age standardization, subjects were divided into 5-year groups, and the 2010 mid-year population from Statistics Korea was used as the reference. Unlike other types of cancer, TC has a very low mortality rate; thus, the annual age-standardized mortality rate by income quintile was derived by combining the data between 2006 and 2010 and the data between 2011 and 2015, respectively, to ensure the stability of TC mortality rates. The incidence and mortality rates of lung cancer and stroke (the negative control outcomes) were derived using age-standardized rates for each income quintile by calendar year between 2006 and 2015. All analyses were performed using SAS version 9.4 (SAS Institute Inc., Cary, NC, USA).

## Results

Table 1 presents the numbers of study subjects aged 20 or over from the database that was used to derive the screening, incidence, postoperative complication, and mortality rates of TC. The number of study subjects in the KCHS data was relatively similar over the years: 225,116 in 2010, 225,188 in 2012, and 224,994 in 2014. The respondents who had received TC screening in the most recent 2 years gradually increased. The number of study subjects in the NHID between 2006 and 2015 has consistently increased, from 35,500,836 in 2006 to 39,696,326 in 2015. From 2006 to 2015, the number of patients with incident TC increased from 15,943 in 2006 to 44,174 in 2012, but then decreased to 25,165 in 2015. The number of patients with postoperative complications of TC surgery increased continually between 2006 and 2010, decreased in 2011, and then increased to its peak of 7694 in 2013. Later, the number of patients gradually decreased, along with TC incidence. In contrast to the incidence and postoperative complication rate of TC, the mortality of TC remained between 300 and 400 every year, without any significant fluctuations between 2006 and 2015. The magnitudes of the screening, incident, postoperative complications, and deaths cases of TC were much higher for women than men. However, the trends were similar to both women and men. The numbers of patients with the negative control outcomes by year are presented in Supplementary Table S3.

**Table 1:** Study subjects (aged 20 or over) from KCHS and NHID in Korea, 2006–2015

Sex	Year	KCHS		NHID			
		No. of study subjects	No. of subjects with TC screening	No. of total population	No. of TC incident cases	No. of subjects with postoperative complications	No. of TC deaths
All	2006	–	–	35,500,836	15,943	3043	322
	2007	–	–	35,881,719	20,564	3565	358
	2008	–	–	36,504,109	26,474	4450	332
	2009	–	–	36,925,146	32,480	5654	342
	2010	225,116	31,701	36,669,946	34,981	6946	344
	2011	–	–	37,725,424	40,081	5640	382
	2012	225,188	42,249	38,139,862	44,174	6937	358
	2013	–	–	38,574,788	42,818	7694	379
	2014	224,994	44,942	38,941,467	31,923	6610	339
	2015	–	–	39,696,326	25,165	4294	331
Women	2006	–	–	18,007,925	13,629	2631	234
	2007	–	–	18,180,997	17,464	3111	253
	2008	–	–	18,507,966	22,294	3851	230
	2009	–	–	18,717,880	27,307	4924	233
	2010	122,432	20,011	18,589,856	28,956	5889	254
	2011	–	–	19,114,446	33,125	4754	269
	2012	123,971	27,322	19,326,295	36,161	5860	235
	2013	–	–	19,541,003	34,362	6269	266
	2014	123,226	29,248	19,717,940	25,529	5431	256
	2015	–	–	20,095,936	19,768	3449	228
Men	2006	–	–	17,492,911	2314	412	88
	2007	–	–	17,700,722	3100	454	105
	2008	–	–	17,996,143	4180	599	102
	2009	–	–	18,207,266	5173	730	109
	2010	102,684	11,690	18,080,090	6025	1057	90
	2011	–	–	18,610,978	6956	886	113
	2012	101,217	14,927	18,813,567	8013	1077	123
	2013	–	–	19,033,785	8456	1425	113
	2014	101,768	15,694	19,223,527	6394	1179	83
	2015	–	–	19,600,390	5397	845	103

*TC* Thyroid cancer, *KCHS* Korea Community Health Survey, *NHID* National Health Insurance Database

**Fig. 1:**
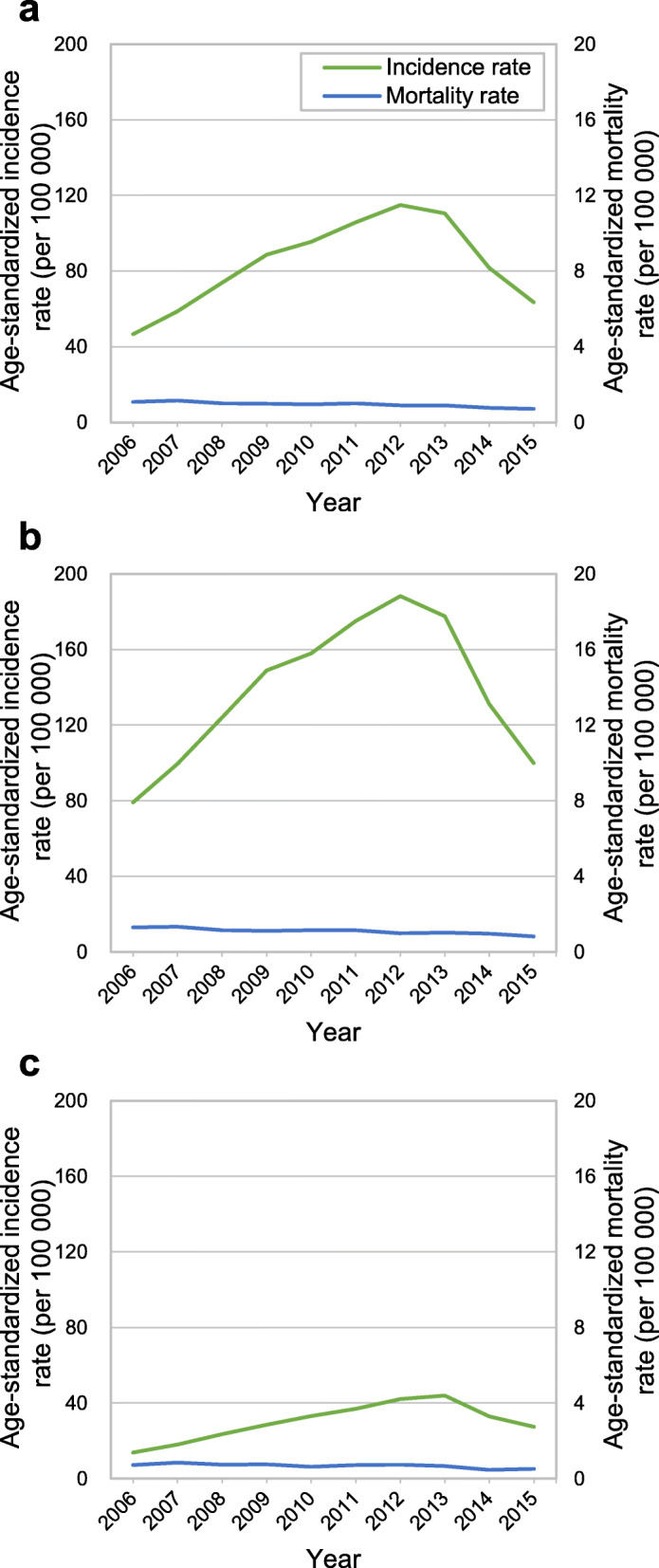
Trends in age-standardized incidence and mortality rates of thyroid cancer in Korea, 2006–2015. **a** women and men combined. **b** women. **c** men

Figure 1 presents the age-standardized incidence and mortality rates of TC in Korea between 2006 and 2015. TC incidence in men and women combined soared from 46.6 to 115.0 per 100,000 from 2006 to 2012, but then plummeted through 2015. TC incidence among women in 2012 was 188.2 per 100,000, reflecting a nearly 2.4-fold increase from 79.1 in 2006. The incidence of TC in men was much lower than in women, but it increased by 3.2 times, from 13.9 per 100,000 in 2006 to 44.0 in 2013. The increasing trend in TC incidence nosedived starting in 2012 among women and starting in 2013 among men. However, the mortality rate remained very low, at approximately 1.0 per 100,000 regardless of sex, and it showed no significant fluctuations between 2006 and 2015 (Supplementary Tables S4 and S5).

Table 2 presents the age-standardized screening, incidence, postoperative complication, and mortality rates of TC by calendar year and income level. In 2010, 2012, and 2014, from 12,562.0 to 18,717.5 adults aged 20 or over per 100,000 had undergone TC screening in the most recent 2 years. Between 2006 and 2015, TC incidence ranged from 46.6 to 115.0 per 100,000, and the postoperative complication rate was 8.9 to 19.7 per 100,000. This indicates that postoperative complications occurred in 14.1 to 20.5% of incident TC cases every year. In contrast, the mortality of TC was stable over time, ranging only from 1.0 per 100,000 between 2006 and 2010 and 0.8 per 100,000 between 2011 and 2015. Every year between 2006 and 2015, the highest income quintile showed high screening and incidence rates of TC. The highest income quintile also showed high rates of postoperative complications associated with TC in most years. However, the mortality rate showed an opposite pattern, as the highest mortality rate occurred in the lowest income quintile.

**Table 2:** Age-standardized screening, incidence, postoperative complication, and mortality rates of TC by income quintiles in Korea, 2006–2015

Age-standardized rate^a^	Year	Overall		Income Q1 (lowest)		Income Q2		Income Q3		Income Q4		Income Q5 (highest)	
Screening prevalence	Mid 2008 -mid 2010	12,562.0 (12,415.9–12,708.0)		8824.2 (8515.1–9133.3)		10,084.2 (9781.0–10,387.5)		11,487.7 (11,180.1–11,795.2)		13,057.9 (12,734.2–13,381.6)		17,675.7 (17,317.5–18,033.9)	
	Mid 2010 -mid 2012	17,449.9 (17,279.2–17,620.6)		11,506.9 (11,161.8–11,852.0)		13,612.7 (13,263.2–13,962.3)		16,305.0 (15,933.0–16,677.0)		18,874.5 (18,494.1–19,254.8)		24,439.6 (24,020.1–24,859.1)	
	Mid 2012 -mid 2014	18,717.5 (18,540.9–18,894.2)		11,977.5 (11,606.0–12,349.0)		14,934.3 (14,574.5–15,294.0)		17,118.8 (16,741.1–17,496.4)		20,138.9 (19,748.2–20,529.6)		26,110.0 (25,675.6–26,544.5)	
Incidence rate	2006	46.6	(45.8–47.3)	36.4	(35–37.8)	37.8	(36.4–39.3)	42.7	(41.2–44.3)	50.2	(48.5–51.9)	66.0	(64.1–68.0)
	2007	58.7	(57.9–59.6)	43.9	(42.3–45.5)	47.3	(45.7–48.9)	55.0	(53.2–56.7)	66.3	(64.3–68.2)	81.6	(79.4–83.7)
	2008	73.7	(72.8–74.6)	53.7	(52.0–55.4)	59.1	(57.3–60.9)	68.0	(66.1–70.0)	83.6	(81.5–85.7)	104.2	(101.9–106.6)
	2009	88.7	(87.7–89.6)	63.6	(61.8–65.4)	73.5	(71.6–75.5)	82.1	(80.0–84.2)	99.8	(97.5–102.1)	124.4	(121.9–127.0)
	2010	95.4	(94.4–96.4)	69.5	(67.6–71.4)	79.6	(77.5–81.6)	90.1	(88.0–92.3)	106.4	(104.0–108.8)	131.4	(128.8–134.1)
	2011	105.8	(104.8–106.9)	78.0	(76.1–80.0)	91.3	(89.2–93.5)	104.0	(101.7–106.3)	119.1	(116.6–121.5)	136.8	(134.2–139.4)
	2012	115.0	(113.9–116)	84.8	(82.8–86.9)	100.1	(97.8–102.3)	113.3	(110.9–115.7)	128.3	(125.8–130.9)	148.3	(145.6–151.0)
	2013	110.5	(109.4–111.5)	81.0	(79.0–83.0)	97.8	(95.6–100.1)	108.0	(105.7–110.4)	125.9	(123.4–128.4)	139.6	(136.9–142.2)
	2014	81.7	(80.8–82.6)	63.4	(61.6–65.1)	75.5	(73.6–77.5)	81.5	(79.5–83.5)	91.2	(89.1–93.3)	97.2	(95.0–99.4)
	2015	63.5	(62.7–64.2)	50.5	(49.0–52.1)	58.4	(56.7–60.1)	63.5	(61.8–65.3)	71.3	(69.5–73.2)	73.4	(71.5–75.3)
Postoperative complication rate	2006	8.9	(8.6–9.2)	6.5	(5.9–7.1)	7.7	(7.0–8.4)	8.4	(7.8–9.1)	9.9	(9.1–10.6)	12.0	(11.2–12.9)
	2007	10.2	(9.8–10.5)	7.9	(7.2–8.5)	8.1	(7.4–8.7)	10.3	(9.6–11.1)	11.7	(10.9–12.5)	12.9	(12.1–13.8)
	2008	12.4	(12.1–12.8)	10.0	(9.3–10.8)	10.6	(9.8–11.4)	12.3	(11.5–13.1)	13.4	(12.5–14.2)	15.8	(14.9–16.7)
	2009	15.5	(15.1–15.9)	12.4	(11.6–13.2)	13.7	(12.9–14.6)	15.4	(14.5–16.3)	16.5	(15.6–17.5)	19.2	(18.2–20.2)
	2010	19.0	(18.5–19.4)	15.2	(14.3–16.1)	17.4	(16.4–18.3)	18.8	(17.8–19.7)	20.2	(19.2–21.3)	23.2	(22.1–24.3)
	2011	14.9	(14.5–15.3)	11.6	(10.9–12.4)	13.9	(13.0–14.7)	14.8	(14.0–15.7)	16.2	(15.3–17.1)	17.8	(16.8–18.7)
	2012	17.9	(17.5–18.4)	15.3	(14.4–16.2)	16.4	(15.5–17.3)	18.1	(17.2–19.1)	19.3	(18.3–20.3)	20.6	(19.6–21.6)
	2013	19.7	(19.2–20.1)	15.9	(15.1–16.8)	18.0	(17.1–19.0)	20.1	(19.1–21.1)	21.7	(20.7–22.8)	22.5	(21.5–23.6)
	2014	16.8	(16.4–17.2)	14.2	(13.4–15.1)	16.4	(15.5–17.3)	17.7	(16.7–18.6)	18.4	(17.5–19.4)	17.1	(16.2–18.0)
	2015	10.7	(10.4–11.0)	9.6	(8.9–10.3)	9.8	(9.2–10.5)	11.3	(10.5–12.0)	12.1	(11.4–12.9)	10.5	(9.8–11.3)
Mortality rate	2006–2010	1.0	(1.0–1.1)	1.3	(1.1–1.4)	0.9	(0.8–1.0)	1.0	(0.9–1.1)	1.0	(0.9–1.1)	1.0	(0.9–1.1)
	2011–2015	0.8	(0.8–0.9)	1.0	(0.9–1.1)	0.8	(0.7–0.9)	0.8	(0.7–0.9)	0.8	(0.7–0.9)	0.7	(0.7–0.8)

^a^Age-standardized rates per 100,000 were presented with 95% confidence intervals

**Fig. 2:**
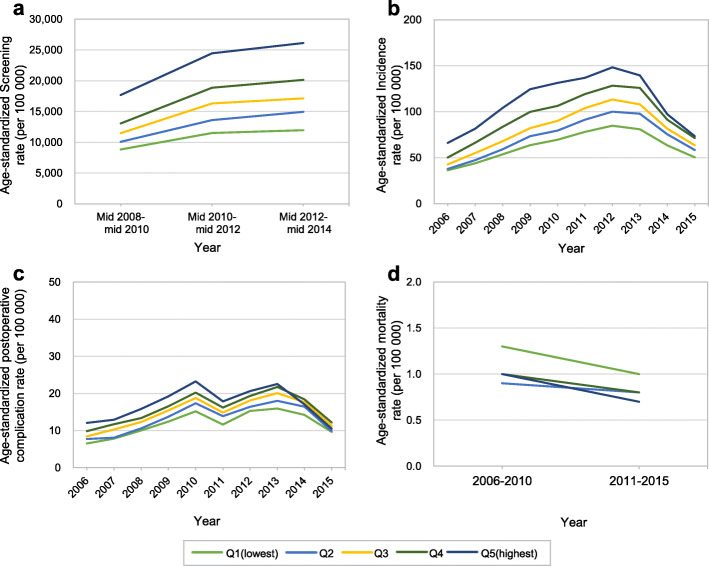
Income differences in screening, incidence, postoperative complications, and mortality of thyroid cancer in South Korea, 2006–2015. **a** screening rate. **b** incidence rate. **c** postoperative complication rate. **d** mortality rate

As shown in Fig. 2, every indicator, except the mortality rate, showed a higher age-standardized rate as income increased (Fig. 2a, b, and c). However, the mortality rate showed the opposite trend (Fig. 2d). The mortality rate of the lowest income quintile was higher than that of the other income quintiles. Furthermore, postoperative complications of TC showed a positive association with income between 2006 and 2013. In 2011, the complication rate decreased in every income group, and then it increased in 2012. The incidence rate began to decline in 2013. The highest income quintile complication rate nosedived, indicating that the highest income participants responded more quickly to the decrease in TC incidence than other income quintiles (Fig. 2c). The annual rates of TC surgery according to income quintiles (Supplementary Figure S1) were also similar to the patterns in TC incidence. On average, about 93% of total incidence cases undergone TC surgery (Supplementary Table S6). The ratios of postoperative complication rates to TC surgery rates were slightly greater in low-income quintiles than high-income quintiles.

**Fig. 3:**
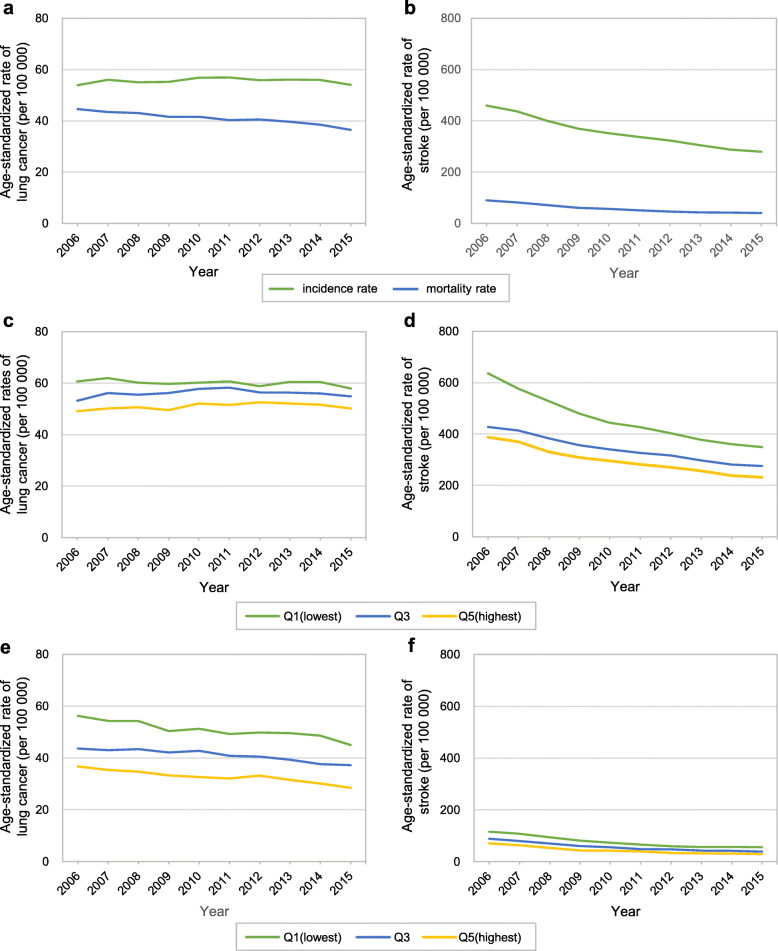
Age-standardized incidence and mortality rates and Income differences therein for negative control outcomes (lung cancer and stroke) in South Korea, 2006–2015. **a** incidence and mortality rates of lung cancer. **b** incidence and mortality rates of stroke. **c** income difference in incidence rates of lung cancer. **d** income difference in incidence rates of stroke. **e** income difference in mortality rates of lung cancer. **f** income difference in mortality rates of stroke

Figure 3 presents the age-standardized incidence and mortality rates of lung cancer and stroke by year and income level. Both lung cancer and stroke showed different patterns of incidence and mortality rates from TC. The incidence and mortality rates of lung cancer and stroke continually decreased. During the observation period, TC showed a rapid increase and decrease, but no similar fluctuation was observed for lung cancer (Fig. 3a) and stroke (Fig. 3b). The incidence of lung cancer showed no clear pattern by calendar year, although the lung cancer mortality rate decreased (Fig. 3a). The incidence and mortality rates of stroke showed a consistent decrease between 2006 and 2015 (Fig. 3b). Every income group also presented a similar pattern. Furthermore, the patterns in incidence rates by income quintiles showed an opposite pattern from that of TC. Whereas TC had a high incidence and low mortality rate in the highest income quintile, for lung cancer and stroke, the lowest income quintile showed the highest rates of incidence and mortality (Fig. 3c, d, e, and f).

## Discussion

The study presented nationally representative rates of TC screening, incidence, postoperative complication, and mortality in Korea. In 2012, the year with the highest incidence of TC, the rate of TC screening within the most recent 2 years was 17,449.9 per 100,000 adults aged 20 or over, while the incidence of TC was 115 per 100,000. In the same year, 17.9 per 100,000 had postoperative complications of TC and 0.9 per 100,000 died of TC. Incident cases of TC potentially included those who had not undergone TC screening, so these results show that TC screening led to a diagnosis of TC in fewer than 1 of 100 examinees. Furthermore, the complication rate in 2012 was 15.6% of incident TC cases (17.9/115*100). Thus, the complication rate was approximately 20 times higher than the mortality rate. It should be noted that this study only examined the rate of TC surgery-specific complications (hypoparathyroidism and vocal cord/fold paralysis) and excluded transient complications within 30 days after surgery. Active surgical interventions for TC are likely to decrease the mortality rate of TC, but this benefit should be balanced with the harm to the population by the postoperative complications of TC surgery. In most cases, the postoperative complications of TC surgery are temporary. However, permanent complications could lead to a low quality of life. Permanent vocal cord paralysis could change an individual’s voice, and patients with permanent hypoparathyroidism need to receive calcium and vitamin D for their entire lifetime. In particular, TC surgery performed to vulnerable, high-risk patients, such as elderly patients with comorbidities, could lead to fairly negative results, including low quality of life, pneumonia, infection, heart complications, and death [13, 23]. The findings of this study, therefore, shed light on the magnitude of the side effects of TC surgery.

The incidence of TC showed very significant fluctuations, but the mortality rate showed no notable change. The incidence of TC between 2011 and 2013 increased more than 2-fold from 2006 in all income brackets. This increased incidence in such a short period is difficult to be explained by dietary and environmental risk factors. The increasing trend of TC incidence was more significant in women than in men (Fig. 1), since ultrasonography for breast cancer screening is often performed jointly with thyroid ultrasonography in Korea. The increase in the incidence of TC and the concomitant lack of change in the mortality rate have been regarded as evidence of the overdiagnosis of TC [4, 24, 25]. In the study, the interpretation of the increase in the incidence of TC as being due to overdiagnosis was further corroborated by data showing that the incidence of TC decreased after 2012, but the mortality rate still showed no changes.

The screening, incidence, surgery rates, and postoperative complication rates of TC were higher in the high-income group than in other income groups. Thyroid cancer screening is conducted as opportunistic health screening and thus might be affected by service users’ financial capability. Greater use of thyroid cancer screening might produce greater incidence rates, surgery rates, and subsequent postoperative complication rates among high-income strata than among low-income strata. However, a higher mortality rate of TC was shown in the lowest income bracket. According to a previous review study, no clear relationship has been shown between the mortality rate of TC and socioeconomic status [26]. The high mortality rate in the lowest income group might be because this income group included Medical Aid recipients with underlying health problems [20, 21]. Previous studies in Korea have reported greater in-hospital mortality rates among Medical Aid recipients [27]. Low quality of care and delayed use of proper health care services might have produce greater TC mortality rates especially among the lowest income group in Korea.

The incidence of TC decreased after 2012 as a result of concerns about TC overdiagnosis; in particular, the incidence decreased rapidly in the high-income group. Even the postoperative complication rate nosedived in the highest income group. In 2015, the incidence of TC postoperative complications in the highest income quintile had decreased to the level of the lowest income quintile. This could be interpreted as indicating that high-income individuals responded more rapidly to concerns about the postoperative complications of TC. In 2011, the number of postoperative complications of TC decreased due to the 2010 revision of the management guidelines on thyroid nodules and TC by the Korean Thyroid Association [8]. Active surveillance associated with this 2010 revision might have contributed to the decline in postoperative complications of TC [28].

Lung cancer and stroke were used as negative control outcomes because these two diseases had a low probability of overdiagnosis. Lung cancer was not included as a component of the cancer screening program in Korea during the study period. No screening program for stroke existed. If the relationships of income with incidence and mortality for these two diseases were similar to the relationships of income with TC incidence and TC mortality, the findings for thyroid cancer are less likely due to overdiagnosis. However, the findings for these two negative control outcomes showed that the incidence and mortality of lung cancer and stroke by income level were different from those of TC, which could corroborate our assertion that the relationship of income with TC incidence and mortality would be due to overdiagnosis. Lung cancer and stroke showed high incidence and mortality rates in low-income individuals. The high incidence and mortality rates of these diseases in low-income individuals could be explained through socioeconomic inequalities in risk factors, including gaps in the smoking rate across income levels [29] and similar gaps in blood pressure [30], as well as inequalities in the use of medical services and the quality thereof.

The study has strengths and limitations. This study employed large nationally representative data in Korea containing information on screening, healthcare utilization, and mortality. Several studies have been conducted on the incidence and mortality rates of TC, but very few studies have assessed the screening and complication rates. To the best of our knowledge, no study has investigated gaps across income levels in relation to the aforementioned indicators. Analyses by pathological subtype of TC were not conducted in this study because we employed NHID to use variables on income and postoperative complications rather than KCCR database. At the time of this study, individual data linkage between NHID and KCCR was not possible. According to a medical records review of TC patients between 1962 and 2009 in Korea, the proportion of small tumors less than 1 cm in the total cases of TC increased from 6.1 to 43.1% [31]. In particular, papillary TC, for which overdiagnosis often occurs, accounted for over 90% of all cases of TC [4, 32]. For example, 97.2% of total TC were papillary thyroid cancer while only 1.8% of total TC were follicular cancer in 2008 [33]. A prior Korean study also showed that the increase in TC from 1999 to 2008 was mainly attributed to the increased incidence of small tumors [33]. In addition, a recent study demonstrated that the TC screening rate was highly associated with the papillary TC incidence rate between 2008 and 2010 across 16 administrative regions of Korea [34]. Even if papillary TC were concentrated in a particular income group, the results on the incidence of TC by income level found in this study would hardly change.

## Conclusions

This study suggests that the considerable fluctuations in the TC incidence over the past decade in Korea were driven by overdiagnosis. The study showed a high screening rate of TC in high-income individuals, leading to high incidence and postoperative complication rates of TC in that group. However, a similar pattern was not shown for the mortality rate. The incidence of lung cancer and stroke, negative control outcomes, did not show a similar pattern to TC by income level. In particular, the recent rapid decrease in the incidence and complication rates in high-income individuals is likely to have been a result of societal concerns over the TC overdiagnosis in Korea. These results provide corroborating evidence that overdiagnosis has caused a considerable proportion of the increase in TC incidence in Korea.

## Supplementary Information

**Additional file 1:** **Table S1.** Annual comparison of numbers of incident cases, crude incidence rate, and age-standardized incidence rate of thyroid cancer between the National Health Information Database (NHID) from the National Health Insurance Service and the Korea Central Cancer Registry (KCCR). **Table S2.** Annual comparison of numbers of incident cases, crude incidence, and age-standardized incidence rate of lung cancer between the National Health Information Database (NHID) from the National Health Insurance Service and the Korea Central Cancer Registry (KCCR). **Table S3.** Study subjects of patients with the negative control outcomes from the National Health Information Database (NHID) in Korea, 2006–2015. **Table S4.** Age-standardized screening prevalence, incidence rate, postoperative complication rate, and mortality rate of thyroid cancer according to income quintiles in women, Korea, 2006–2015. **Table S5.** Age-standardized screening prevalence, incidence rate, postoperative complication rate, and mortality rate of thyroid cancer according to income quintiles in men, Korea, 2006–2015. **Table S6.** Ratios of thyroid cancer surgery to thyroid cancer incidence according to income quintiles and ratios of postoperative complications to thyroid cancer surgery according to income quintiles, Korea, 2006–2015

**Additional file 2:** **Figure S1.** Income differences in age-standardized surgery rate of thyroid cancer in Korea. (PPTX 79 kb)

## Data Availability

The Korea Community Health Survey (KCHS) data are publicly available. The National Health Information Database (NHID) that support the finding of this study are available from the Big Data Steering Department of the National Health Insurance Service, but restrictions apply to the availability of these data, which were used under license for the current study, and so are not publicly available. The authors used the aggregate population and death data without personal identification numbers. De-identified aggregate data are available from the corresponding author after permissions are obtained from the Big Data Steering Department of the National Health Insurance Service in Korea.

## Abbreviations

TC: Thyroid cancer
KCHS: Korea Community Health Survey
NHID: National Health Information Database
NHIS: National Health Insurance Service
KCCR: Korea Central Cancer Registry
NHI: National Health Insurance (NHI)
