# Supplementary material for: Income differences in screening, incidence, postoperative complications, and mortality of thyroid cancer in South Korea: a national population-based time trend study
Source: BMC Cancer. 2020 Nov 11;20:1096. doi: 10.1186/s12885-020-07597-4 (PMC7661203; doi:10.1186/s12885-020-07597-4)
Supplement: Supplementary file 1 — Additional file 1: Table S1. Annual comparison of numbers of incident cases, crude incidence rate, and age-standardized incidence rate of thyroid cancer between the National Health Information Database (NHID) from the National Health Insurance Service and the Korea Central Cancer Registry (KCCR). Table S2. Annual comparison of numbers of incident cases, crude incidence, and age-standardized incidence rate of lung cancer between the National Health Information Database (NHID) from the National Health Insurance Service and the Korea Central Cancer Registry (KCCR). Table S3. Study subjects of patients with the negative control outcomes from the National Health Information Database (NHID) in Korea, 2006–2015. Table S4. Age-standardized screening prevalence, incidence rate, postoperative complication rate, and mortality rate of thyroid cancer according to income quintiles in women, Korea, 2006–2015. Table S5. Age-standardized screening prevalence, incidence rate, postoperative complication rate, and mortality rate of thyroid cancer according to income quintiles in men, Korea, 2006–2015. Table S6. Ratios of thyroid cancer surgery to thyroid cancer incidence according to income quintiles and ratios of postoperative complications to thyroid cancer surgery according to income quintiles, Korea, 2006–2015 [file 12885_2020_7597_MOESM1_ESM.docx]

**Table S1.** Annual comparison of numbers of incident cases, crude incidence rate, and age-standardized incidence rate of thyroid cancer between the National Health Information Database (NHID) from the National Health Insurance Service and the Korea Central Cancer Registry (KCCR)

| **Year** | **NHID** | | | **KCCR** | | | **NHID/KCCR** | | |
| --- | --- | --- | --- | --- | --- | --- | --- | --- | --- |
|  | **No. of incident cases** | **Crude incidence rate**  **(per 100000)** | **Age-standardized incidence rate**  **(per 100000)** | **No. of incident cases** | **Crude incidence rate**  **(per 100000)** | **Age-standardized incidence rate**  **(per 100000)** | **No. of incident cases** | **Age-standardized incidence rate** |  |
| 2006 | 15943 | 44.9 | 46.6 | 16053 | 43.9 | 45.4 | 0.99 | 1.03 |  |
| 2007 | 20564 | 57.3 | 58.7 | 21158 | 57.2 | 58.6 | 0.97 | 1.00 |  |
| 2008 | 26474 | 72.5 | 73.7 | 27170 | 72.6 | 73.7 | 0.97 | 1.00 |  |
| 2009 | 32480 | 88.0 | 88.7 | 32351 | 85.5 | 86.1 | 1.00 | 1.03 |  |
| 2010 | 34981 | 95.4 | 95.4 | 36579 | 95.7 | 95.7 | 0.96 | 1.00 |  |
| 2011 | 40081 | 106.2 | 105.8 | 41093 | 106.3 | 105.7 | 0.98 | 1.00 |  |
| 2012 | 44174 | 115.8 | 115.0 | 44428 | 113.5 | 112.7 | 0.99 | 1.02 |  |
| 2013 | 42818 | 111.0 | 110.5 | 42703 | 107.9 | 107.4 | 1.00 | 1.03 |  |
| 2014 | 31923 | 82.0 | 81.7 | 30873 | 77.1 | 76.9 | 1.03 | 1.06 |  |
| 2015 | 25165 | 63.4 | 63.5 | 24848 | 61.3 | 61.5 | 1.01 | 1.03 |  |

*KCCR* Korea Central Cancer Registry; *NHID* National Health Insurance Database

**Table S2.** Annual comparison of numbers of incident cases, crude incidence rate, and age-standardized incidence rate of lung cancer between the National Health Information Database (NHID) from the National Health Insurance Service and the Korea Central Cancer Registry (KCCR)

| **Year** | **NHID** | | | **KCCR** | | | **NHID/KCCR** | | |
| --- | --- | --- | --- | --- | --- | --- | --- | --- | --- |
|  | **No. of incident cases** | **Crude incidence rate**  **(per 100000)** | **Age-standardized incidence rate**  **(per 100000)** | **No. of incident cases** | **Crude incidence rate**  **(per 100000)** | **Age-standardized incidence rate**  **(per 100000)** | **No. of incident cases** | **Age-standardized incidence rate** |  |
| 2006 | 16656 | 46.9 | 54.0 | 0.94 | 48.4 | 55.3 | 0.94 | 0.98 |  |
| 2007 | 17935 | 50.0 | 56.0 | 0.97 | 49.9 | 55.1 | 0.97 | 1.02 |  |
| 2008 | 18581 | 50.9 | 55.1 | 0.97 | 51.2 | 54.7 | 0.97 | 1.01 |  |
| 2009 | 19439 | 52.6 | 55.3 | 0.97 | 53.0 | 54.8 | 0.97 | 1.01 |  |
| 2010 | 20641 | 56.3 | 56.8 | 0.97 | 55.6 | 55.6 | 0.97 | 1.02 |  |
| 2011 | 21798 | 57.8 | 57.0 | 0.98 | 57.4 | 55.7 | 0.98 | 1.02 |  |
| 2012 | 22271 | 58.4 | 55.9 | 0.99 | 57.4 | 53.9 | 0.99 | 1.04 |  |
| 2013 | 23414 | 60.7 | 56.1 | 1.00 | 59.3 | 53.8 | 1.00 | 1.04 |  |
| 2014 | 24249 | 62.3 | 55.9 | 1.00 | 60.5 | 53.4 | 1.00 | 1.05 |  |
| 2015 | 24604 | 62.0 | 54.1 | 1.01 | 59.9 | 51.3 | 1.01 | 1.06 |  |

*KCCR* Korea Central Cancer Registry; *NHID* National Health Insurance Database

**Table S3.** Study subjects of patients with the negative control outcomes from the National Health Information Database (NHID) in Korea, 2006-2015

| **Year** | **Lung cancer** | | | | | | **Stroke** | | | | | |
| --- | --- | --- | --- | --- | --- | --- | --- | --- | --- | --- | --- | --- |
|  | **No. of incident cases** | **Crude incidence rate  (per 100000)** | **Age-standardized incidence rate  (per 100000)** | **No. of deaths** | **Crude**  **death rate  (per 100000)** | **Age-standardized death rate (per 100000)** | **No. of incident cases** | **Crude incidence rate  (per 100000)** | **Age-standardized incidence rate  (per 100000)** | **No. of deaths** | **Crude death rate  (per 100000)** | **Age-standardized death rate  (per 100000)** |
| 2006 | 16656 | 46.9 | 54.0 | 13512 | 38.1 | 44.6 | 115301 | 324.8 | 459.6 | 21651 | 61.0 | 89.9 |
| 2007 | 17935 | 50.5 | 56.0 | 13721 | 38.6 | 43.5 | 113714 | 320.3 | 436.5 | 20562 | 57.9 | 81.7 |
| 2008 | 18581 | 52.3 | 55.1 | 14375 | 40.5 | 43.1 | 109725 | 309.1 | 399.6 | 19028 | 53.6 | 71.2 |
| 2009 | 19439 | 54.8 | 55.3 | 14528 | 40.9 | 41.6 | 106166 | 299.1 | 369.7 | 17140 | 48.3 | 60.7 |
| 2010 | 20641 | 58.1 | 56.8 | 15065 | 42.4 | 41.6 | 104390 | 294.0 | 351.3 | 16711 | 47.1 | 56.6 |
| 2011 | 21798 | 61.4 | 57.0 | 15444 | 43.5 | 40.3 | 105670 | 297.7 | 337.1 | 16055 | 45.2 | 51.1 |
| 2012 | 22271 | 62.7 | 55.9 | 16249 | 45.8 | 40.6 | 105588 | 297.4 | 323.0 | 15221 | 42.9 | 46.1 |
| 2013 | 23414 | 66.0 | 56.1 | 16733 | 47.1 | 39.6 | 104148 | 293.4 | 304.4 | 15065 | 42.4 | 43.1 |
| 2014 | 24249 | 68.3 | 55.9 | 16960 | 47.8 | 38.5 | 102444 | 288.6 | 287.8 | 15544 | 43.8 | 42.4 |
| 2015 | 24604 | 69.3 | 54.1 | 16945 | 47.7 | 36.5 | 104382 | 294.0 | 279.6 | 15955 | 44.9 | 40.8 |

**Table S4.** Age-standardized screening prevalence, incidence rate, postoperative complication rate, and mortality rate of thyroid cancer according to income quintiles in women, Korea, 2006-2015

| **Rate^a^** | **Year** | **Overall** | **Income Q1**  **(lowest)** | **Income Q2** | **Income Q3** | **Income Q4** | **Income Q5**  **(highest)** |
| --- | --- | --- | --- | --- | --- | --- | --- |
| Screening  Prevalence | Mid 2008-  mid 2010 | 15214.0 (14996.0 to 15432.0) | 11203.7 (10730.6 to 11676.8) | 12435.8 (11982.8 to 12888.9) | 14183.9 (13724.0 to 14643.9) | 15917.4 (15420.7 to 16414.1) | 20392.3 (19874.8 to 20909.8) |
|  | Mid 2010-  mid 2012 | 21350.4 (21095.1 to 21605.7) | 14977.3 (14449.3 to 15505.2) | 17227.7 (16693.3 to 17762.1) | 20229.4 (19673.0 to 20785.9) | 22728.7 (22164.2 to 23293.2) | 28927.9 (28306.4 to 29549.5) |
|  | Mid 2012-  mid 2014 | 22982.1 (22716.8 to 23247.4) | 15677.1 (15090.2 to 16264.1) | 18790.9 (18250.2 to 19331.6) | 21182.5 (20604.6 to 21760.4) | 24764.0 (24171.9 to 25356.0) | 30803.9 (30165.5 to 31442.3) |
| Incidence  rate | 2006 | 79.1 (77.8 to 80.4) | 63.5 (60.8 to 66.2) | 65.9 (63.2 to 68.6) | 73.8 (70.9 to 76.7) | 84.2 (81.2 to 87.3) | 108.5 (104.9 to 112.0) |
|  | 2007 | 99.5 (98.0 to 101.0) | 77.0 (74.1 to 79.9) | 81.7 (78.7 to 84.7) | 96.0 (92.8 to 99.3) | 111.0 (107.5 to 114.5) | 131.9 (128.1 to 135.7) |
|  | 2008 | 123.9 (122.2 to 125.5) | 94.1 (90.9 to 97.3) | 102.9 (99.6 to 106.2) | 115.9 (112.4 to 119.4) | 138.0 (134.2 to 141.9) | 168.4 (164.2 to 172.7) |
|  | 2009 | 148.9 (147.1 to 150.6) | 113.7 (110.2 to 117.1) | 129.5 (125.8 to 133.3) | 140.5 (136.6 to 144.3) | 164.4 (160.2 to 168.5) | 196.4 (191.9 to 201.0) |
|  | 2010 | 158.0 (156.1 to 159.8) | 122.3 (118.7 to 125.9) | 137.4 (133.6 to 141.2) | 151.9 (147.9 to 155.9) | 172.2 (167.9 to 176.4) | 206.1 (201.5 to 210.8) |
|  | 2011 | 175.0 (173.1 to 176.9) | 136.7 (133.0 to 140.4) | 157.7 (153.7 to 161.7) | 174.0 (169.8 to 178.2) | 191.2 (186.8 to 195.6) | 215.6 (210.9 to 220.3) |
|  | 2012 | 188.2 (186.3 to 190.2) | 148.7 (144.9 to 152.6) | 170.9 (166.8 to 175.1) | 187.1 (182.7 to 191.4) | 204.1 (199.6 to 208.7) | 230.4 (225.6 to 235.2) |
|  | 2013 | 177.6 (175.7 to 179.5) | 140.0 (136.2 to 143.7) | 167.5 (163.4 to 171.6) | 175.9 (171.7 to 180.1) | 195.9 (191.5 to 200.4) | 208.6 (204.0 to 213.2) |
|  | 2014 | 131.1 (129.5 to 132.7) | 108.2 (104.9 to 111.5) | 127.0 (123.5 to 130.6) | 132.0 (128.4 to 135.7) | 141.9 (138.1 to 145.7) | 146.3 (142.5 to 150.2) |
|  | 2015 | 99.9 (98.5 to 101.3) | 85.7 (82.8 to 88.6) | 97.6 (94.5 to 100.7) | 100.6 (97.4 to 103.7) | 109.4 (106.1 to 112.7) | 106.2 (102.9 to 109.5) |
| Postoperative  complication  rate | 2006 | 15.3 (14.7 to 15.9) | 11.4 (10.3 to 12.5) | 13.4 (12.1 to 14.6) | 14.9 (13.6 to 16.2) | 16.8 (15.5 to 18.2) | 20.1 (18.6 to 21.6) |
|  | 2007 | 17.7 (17.1 to 18.3) | 14.0 (12.8 to 15.3) | 13.9 (12.7 to 15.1) | 18.6 (17.1 to 20.0) | 20.1 (18.6 to 21.6) | 22.0 (20.4 to 23.5) |
|  | 2008 | 21.5 (20.8 to 22.1) | 17.8 (16.4 to 19.2) | 19.0 (17.6 to 20.5) | 21.3 (19.8 to 22.9) | 22.8 (21.2 to 24.3) | 26.4 (24.7 to 28.0) |
|  | 2009 | 26.8 (26.1 to 27.6) | 22.1 (20.6 to 23.7) | 24.6 (23.0 to 26.2) | 27.6 (25.9 to 29.3) | 27.9 (26.2 to 29.6) | 31.8 (30.0 to 33.7) |
|  | 2010 | 32.1 (31.2 to 32.9) | 26.8 (25.1 to 28.4) | 30.1 (28.3 to 31.9) | 32.4 (30.5 to 34.2) | 33.9 (32.0 to 35.8) | 37.2 (35.2 to 39.2) |
|  | 2011 | 24.9 (24.2 to 25.7) | 20.5 (19.1 to 22.0) | 24.1 (22.5 to 25.7) | 25.3 (23.7 to 26.9) | 25.9 (24.3 to 27.5) | 28.9 (27.2 to 30.6) |
|  | 2012 | 30.2 (29.5 to 31.0) | 27.0 (25.4 to 28.6) | 28.5 (26.8 to 30.2) | 30.8 (29.0 to 32.5) | 31.9 (30.2 to 33.7) | 33.0 (31.2 to 34.8) |
|  | 2013 | 31.9 (31.1 to 32.7) | 27.6 (26.0 to 29.3) | 31.0 (29.2 to 32.7) | 33.3 (31.5 to 35.1) | 34.1 (32.3 to 36.0) | 33.7 (31.9 to 35.5) |
|  | 2014 | 27.6 (26.8 to 28.3) | 24.8 (23.3 to 26.4) | 28.4 (26.7 to 30.1) | 29.9 (28.2 to 31.6) | 28.7 (27.0 to 30.4) | 26.0 (24.4 to 27.6) |
|  | 2015 | 17.2 (16.6 to 17.8) | 16.5 (15.2 to 17.7) | 16.8 (15.5 to 18.0) | 18.0 (16.7 to 19.3) | 18.7 (17.3 to 20.1) | 16.0 (14.8 to 17.3) |
| Mortality  rate | 2006-2010 | 1.2 (1.1 to 1.3) | 1.5 (1.4 to 1.7) | 1.0 (0.9 to 1.2) | 1.1 (1.0 to 1.3) | 1.1 (1.0 to 1.3) | 1.2 (1.1 to 1.4) |
|  | 2011-2015 | 1.0 (0.9 to 1.0) | 1.2 (1.0 to 1.3) | 0.9 (0.8 to 1.0) | 1.0 (0.8 to 1.1) | 1.0 (0.9 to 1.1) | 0.9 (0.8 to 1.0) |
|  | 2006 | 1.3 (1.1 to 1.5) | 1.4 (1.0 to 1.8) | 1.1 (0.8 to 1.5) | 1.4 (1.0 to 1.8) | 1.1 (0.8 to 1.5) | 1.5 (1.1 to 1.9) |
|  | 2007 | 1.3 (1.2 to 1.5) | 2.0 (1.6 to 2.4) | 1.3 (0.9 to 1.6) | 1.0 (0.7 to 1.4) | 1.2 (0.9 to 1.6) | 1.2 (0.9 to 1.6) |
|  | 2008 | 1.2 (1.0 to 1.3) | 1.5 (1.1 to 1.9) | 1.0 (0.7 to 1.3) | 1.1 (0.8 to 1.4) | 1.2 (0.8 to 1.5) | 1.1 (0.8 to 1.4) |
|  | 2009 | 1.1 (1.0 to 1.3) | 1.2 (0.9 to 1.5) | 1.0 (0.7 to 1.3) | 1.0 (0.7 to 1.3) | 1.1 (0.8 to 1.4) | 1.3 (0.9 to 1.6) |
|  | 2010 | 1.2 (1.0 to 1.3) | 1.5 (1.2 to 1.9) | 1.0 (0.7 to 1.3) | 1.1 (0.8 to 1.5) | 1.2 (0.9 to 1.5) | 1.0 (0.7 to 1.3) |
|  | 2011 | 1.2 (1.0 to 1.3) | 1.6 (1.2 to 1.9) | 0.9 (0.6 to 1.2) | 1.2 (0.9 to 1.5) | 1.2 (0.8 to 1.5) | 1.0 (0.7 to 1.3) |
|  | 2012 | 1.0 (0.9 to 1.1) | 1.0 (0.7 to 1.3) | 1.0 (0.7 to 1.3) | 1.0 (0.7 to 1.2) | 1.0 (0.7 to 1.3) | 1.0 (0.7 to 1.2) |
|  | 2013 | 1.0 (0.9 to 1.1) | 1.0 (0.7 to 1.3) | 0.8 (0.6 to 1.1) | 1.2 (0.9 to 1.5) | 1.0 (0.7 to 1.3) | 1.1 (0.8 to 1.4) |
|  | 2014 | 1.0 (0.8 to 1.1) | 1.2 (0.9 to 1.5) | 1.0 (0.7 to 1.3) | 0.9 (0.6 to 1.2) | 1.0 (0.7 to 1.2) | 0.8 (0.6 to 1.1) |
|  | 2015 | 0.8 (0.7 to 0.9) | 1.1 (0.8 to 1.4) | 0.7 (0.5 to 1.0) | 0.7 (0.5 to 1.0) | 0.9 (0.6 to 1.1) | 0.8 (0.5 to 1.0) |

^a^ Age-standardized rates per 100000 were presented with 95% confidence intervals.

**Table S5.** Age-standardized screening prevalence, incidence rate, postoperative complication rate, and mortality rate of thyroid cancer according to income quintiles in men, Korea, 2006-2015

| **Rate^a^** | **Year** | **Overall** | **Income Q1**  **(lowest)** | **Income Q2** | **Income Q3** | **Income Q4** | **Income Q5**  **(highest)** |
| --- | --- | --- | --- | --- | --- | --- | --- |
| Screening  prevalence | Mid 2008-  mid 2010 | 9520.9 (9330.2 to 9711.7) | 6203.5 (5809.1 to 6597.9) | 7286.6 (6894.7 to 7678.5) | 8294.1 (7897.5 to 8690.6) | 10048.2 (9629.4 to 10467.0) | 14431.4 (13943.0 to 14919.8) |
|  | Mid 2010-  mid 2012 | 12827.4 (12607.4 to 13047.3) | 7308.4 (6886.6 to 7730.3) | 9429.5 (8995.3 to 9863.6) | 11455.8 (10981.1 to 11930.4) | 14276.3 (13780.4 to 14772.1) | 19256.0 (18701.4 to 19810.6) |
|  | Mid 2012-  mid 2014 | 13652.6 (13427.5 to 13877.7) | 7710.8 (7274.3 to 8147.2) | 10150.6 (9686.7 to 10614.5) | 12326.7 (11847.1 to 12806.3) | 14625.9 (14131.4 to 15120.5) | 20487.2 (19911.3 to 21063.1) |
| Incidence  rate | 2006 | 13.9 (13.3 to 14.5) | 8.8 (7.7 to 9.8) | 9.7 (8.7 to 10.8) | 11.6 (10.4 to 12.8) | 16.0 (14.7 to 17.4) | 23.4 (21.8 to 25.1) |
|  | 2007 | 18.1 (17.4 to 18.7) | 10.5 (9.4 to 11.6) | 13.0 (11.7 to 14.2) | 14.3 (13.0 to 15.6) | 21.6 (20.0 to 23.2) | 31.2 (29.3 to 33.1) |
|  | 2008 | 23.6 (22.9 to 24.4) | 13.4 (12.1 to 14.6) | 15.4 (14.1 to 16.7) | 20.2 (18.7 to 21.7) | 29.2 (27.4 to 31.0) | 40.1 (38.0 to 42.2) |
|  | 2009 | 28.5 (27.7 to 29.3) | 13.5 (12.3 to 14.7) | 17.7 (16.3 to 19.1) | 23.7 (22.1 to 25.3) | 35.3 (33.4 to 37.3) | 52.5 (50.1 to 54.8) |
|  | 2010 | 33.1 (32.3 to 34.0) | 16.9 (15.6 to 18.3) | 22.0 (20.5 to 23.5) | 28.5 (26.7 to 30.2) | 41.1 (39.0 to 43.2) | 57.1 (54.7 to 59.6) |
|  | 2011 | 37.0 (36.1 to 37.9) | 19.8 (18.4 to 21.2) | 25.2 (23.6 to 26.8) | 34.3 (32.4 to 36.2) | 47.4 (45.2 to 49.6) | 58.3 (55.9 to 60.7) |
|  | 2012 | 42.1 (41.2 to 43.0) | 21.4 (19.9 to 22.9) | 29.5 (27.8 to 31.3) | 40.0 (38.0 to 42.1) | 52.9 (50.6 to 55.2) | 66.6 (64.0 to 69.2) |
|  | 2013 | 44.0 (43.0 to 44.9) | 22.5 (21.0 to 24.1) | 28.9 (27.1 to 30.6) | 40.8 (38.8 to 42.8) | 56.5 (54.2 to 58.9) | 71.1 (68.5 to 73.8) |
|  | 2014 | 32.9 (32.1 to 33.7) | 19.4 (18.0 to 20.8) | 24.2 (22.6 to 25.7) | 31.6 (29.8 to 33.3) | 41.0 (38.9 to 43.0) | 48.4 (46.2 to 50.6) |
|  | 2015 | 27.4 (26.7 to 28.2) | 15.7 (14.5 to 17.0) | 19.8 (18.4 to 21.2) | 26.9 (25.3 to 28.6) | 33.7 (31.9 to 35.6) | 41.0 (39.0 to 43.1) |
| Postoperative  complication  rate | 2006 | 2.5 (2.2 to 2.7) | 1.6 (1.2 to 2.1) | 2.0 (1.5 to 2.5) | 1.9 (1.4 to 2.4) | 2.9 (2.3 to 3.5) | 4.0 (3.3 to 4.7) |
|  | 2007 | 2.6 (2.4 to 2.9) | 1.7 (1.3 to 2.1) | 2.2 (1.7 to 2.8) | 2.1 (1.7 to 2.6) | 3.3 (2.6 to 3.9) | 3.8 (3.1 to 4.5) |
|  | 2008 | 3.4 (3.1 to 3.6) | 2.2 (1.7 to 2.7) | 2.1 (1.6 to 2.6) | 3.2 (2.6 to 3.8) | 4.1 (3.4 to 4.8) | 5.2 (4.5 to 6.0) |
|  | 2009 | 4.0 (3.7 to 4.3) | 2.5 (2.0 to 3.0) | 2.8 (2.3 to 3.3) | 3.2 (2.6 to 3.7) | 5.1 (4.4 to 5.9) | 6.5 (5.7 to 7.4) |
|  | 2010 | 5.8 (5.5 to 6.2) | 3.6 (3.0 to 4.2) | 4.6 (3.9 to 5.3) | 5.2 (4.4 to 5.9) | 6.6 (5.7 to 7.4) | 9.2 (8.3 to 10.2) |
|  | 2011 | 4.7 (4.4 to 5.0) | 2.7 (2.1 to 3.2) | 3.5 (2.9 to 4.1) | 4.2 (3.6 to 4.9) | 6.3 (5.5 to 7.1) | 6.6 (5.8 to 7.5) |
|  | 2012 | 5.6 (5.3 to 6.0) | 3.7 (3.1 to 4.3) | 4.2 (3.5 to 4.8) | 5.5 (4.7 to 6.2) | 6.7 (5.9 to 7.5) | 8.2 (7.3 to 9.1) |
|  | 2013 | 7.4 (7.0 to 7.8) | 4.4 (3.7 to 5.0) | 5.1 (4.4 to 5.8) | 6.9 (6.0 to 7.7) | 9.2 (8.3 to 10.2) | 11.3 (10.2 to 12.4) |
|  | 2014 | 6.0 (5.7 to 6.4) | 3.8 (3.2 to 4.4) | 4.5 (3.8 to 5.2) | 5.6 (4.8 to 6.3) | 8.1 (7.2 to 9.0) | 8.1 (7.2 to 9.0) |
|  | 2015 | 4.2 (3.9 to 4.5) | 2.8 (2.3 to 3.4) | 3.0 (2.5 to 3.6) | 4.6 (3.9 to 5.3) | 5.6 (4.9 to 6.3) | 5.1 (4.4 to 5.8) |
| Mortality  rate | 2006-2010 | 0.7 (0.7 to 0.8) | 0.8 (0.6 to 1.0) | 0.7 (0.5 to 0.8) | 0.8 (0.6 to 0.9) | 0.7 (0.6 to 0.9) | 0.7 (0.6 to 0.9) |
|  | 2011-2015 | 0.6 (0.6 to 0.7) | 0.7 (0.6 to 0.9) | 0.7 (0.6 to 0.8) | 0.6 (0.5 to 0.7) | 0.6 (0.5 to 0.7) | 0.5 (0.4 to 0.6) |
|  | 2006 | 0.7 (0.6 to 0.9) | 0.9 (0.5 to 1.3) | 0.7 (0.4 to 1.0) | 0.7 (0.3 to 1.0) | 0.6 (0.3 to 1.0) | 0.7 (0.4 to 1.0) |
|  | 2007 | 0.9 (0.7 to 1.0) | 1.0 (0.6 to 1.4) | 0.8 (0.4 to 1.2) | 0.8 (0.4 to 1.2) | 1.1 (0.6 to 1.6) | 0.6 (0.3 to 1.0) |
|  | 2008 | 0.7 (0.6 to 0.9) | 1.0 (0.6 to 1.4) | 0.6 (0.3 to 0.9) | 0.9 (0.5 to 1.2) | 0.7 (0.4 to 1.1) | 0.5 (0.3 to 0.8) |
|  | 2009 | 0.8 (0.6 to 0.9) | 0.7 (0.4 to 1.1) | 0.7 (0.4 to 1.0) | 0.8 (0.5 to 1.1) | 0.7 (0.3 to 1.0) | 1.0 (0.6 to 1.3) |
|  | 2010 | 0.6 (0.5 to 0.8) | 0.5 (0.2 to 0.7) | 0.6 (0.3 to 0.9) | 0.8 (0.5 to 1.2) | 0.5 (0.2 to 0.8) | 0.8 (0.5 to 1.2) |
|  | 2011 | 0.7 (0.6 to 0.9) | 0.8 (0.5 to 1.1) | 1.0 (0.6 to 1.4) | 0.6 (0.3 to 0.9) | 0.7 (0.4 to 1.0) | 0.6 (0.3 to 0.9) |
|  | 2012 | 0.7 (0.6 to 0.9) | 1.0 (0.7 to 1.4) | 0.8 (0.5 to 1.1) | 0.8 (0.5 to 1.1) | 0.6 (0.3 to 0.8) | 0.7 (0.4 to 0.9) |
|  | 2013 | 0.7 (0.5 to 0.8) | 0.8 (0.5 to 1.1) | 0.7 (0.4 to 1.0) | 0.8 (0.5 to 1.1) | 0.7 (0.4 to 0.9) | 0.4 (0.2 to 0.7) |
|  | 2014 | 0.5 (0.4 to 0.6) | 0.6 (0.3 to 0.9) | 0.5 (0.3 to 0.7) | 0.3 (0.2 to 0.5) | 0.5 (0.2 to 0.7) | 0.5 (0.2 to 0.7) |
|  | 2015 | 0.5 (0.4 to 0.6) | 0.6 (0.3 to 0.8) | 0.6 (0.3 to 0.8) | 0.5 (0.3 to 0.8) | 0.6 (0.4 to 0.9) | 0.4 (0.2 to 0.6) |

^a^ Age-standardized rates per 100000 were presented with 95% confidence intervals.

**Table S6.** Ratios of thyroid cancer surgery to thyroid cancer incidence according to income quintiles and ratios of postoperative complications to thyroid cancer surgery according to income quintiles, Korea, 2006-2015

|  | Income Q1 (lowest) | Income Q2 | Income Q3 | Income Q4 | Income Q5 (highest) | Average ratios |
| --- | --- | --- | --- | --- | --- | --- |
| Ratios of thyroid cancer surgery to thyroid cancer incidence^a^ | | | | | |  |
| 2006 | 0.84 | 0.96 | 0.96 | 0.97 | 0.97 | 0.94 |
| 2007 | 0.92 | 0.93 | 0.95 | 0.94 | 0.95 | 0.94 |
| 2008 | 0.93 | 0.95 | 0.94 | 0.95 | 0.94 | 0.94 |
| 2009 | 0.93 | 0.93 | 0.93 | 0.92 | 0.90 | 0.92 |
| 2010 | 0.92 | 0.92 | 0.91 | 0.91 | 0.88 | 0.91 |
| 2011 | 0.93 | 0.93 | 0.93 | 0.93 | 0.91 | 0.93 |
| 2012 | 0.94 | 0.94 | 0.95 | 0.92 | 0.92 | 0.93 |
| 2013 | 0.95 | 0.94 | 0.93 | 0.93 | 0.92 | 0.93 |
| 2014 | 0.93 | 0.94 | 0.94 | 0.94 | 0.93 | 0.94 |
| 2015 | 0.90 | 0.91 | 0.92 | 0.90 | 0.88 | 0.90 |
| Average ratios | 0.92 | 0.94 | 0.94 | 0.93 | 0.92 | 0.93 |
| Ratios of post-operative complications to thyroid cancer surgery^b^ | | | | | | |
| 2006 | 0.21 | 0.21 | 0.20 | 0.20 | 0.19 | 0.20 |
| 2007 | 0.19 | 0.18 | 0.20 | 0.19 | 0.17 | 0.19 |
| 2008 | 0.20 | 0.19 | 0.19 | 0.17 | 0.16 | 0.18 |
| 2009 | 0.21 | 0.20 | 0.20 | 0.18 | 0.17 | 0.19 |
| 2010 | 0.24 | 0.24 | 0.23 | 0.21 | 0.20 | 0.22 |
| 2011 | 0.16 | 0.16 | 0.15 | 0.15 | 0.14 | 0.15 |
| 2012 | 0.19 | 0.17 | 0.17 | 0.16 | 0.15 | 0.17 |
| 2013 | 0.21 | 0.20 | 0.20 | 0.19 | 0.18 | 0.19 |
| 2014 | 0.24 | 0.23 | 0.23 | 0.22 | 0.19 | 0.22 |
| 2015 | 0.21 | 0.19 | 0.19 | 0.19 | 0.16 | 0.19 |
| Average ratios | 0.21 | 0.20 | 0.20 | 0.18 | 0.17 | 0.19 |

^a^ Ratios of thyroid cancer surgery to thyroid cancer incidence were calculated using age-standardized surgery rates (numerator, Figure S1) and age-standardized thyroid cancer incidence rates (denominator, Table 2).

^b^ Ratios of post-operative complications to thyroid cancer surgery were calculated using age-standardized post-operative complication rates (numerator, Table 2) and age-standardized surgery rates (denominator, Figure S1).
