# Supplementary material for: Income differences in screening, incidence, postoperative complications, and mortality of thyroid cancer in South Korea: a national population-based time trend study
Source: BMC Cancer. 2020 Nov 11;20:1096. doi: 10.1186/s12885-020-07597-4 (PMC7661203; doi:10.1186/s12885-020-07597-4)
Supplement: Supplementary file 2 — Additional file 2: Figure S1. Income differences in age-standardized surgery rate of thyroid cancer in Korea. (PPTX 79 kb) [file 12885_2020_7597_MOESM2_ESM.pptx]

## Slide 1
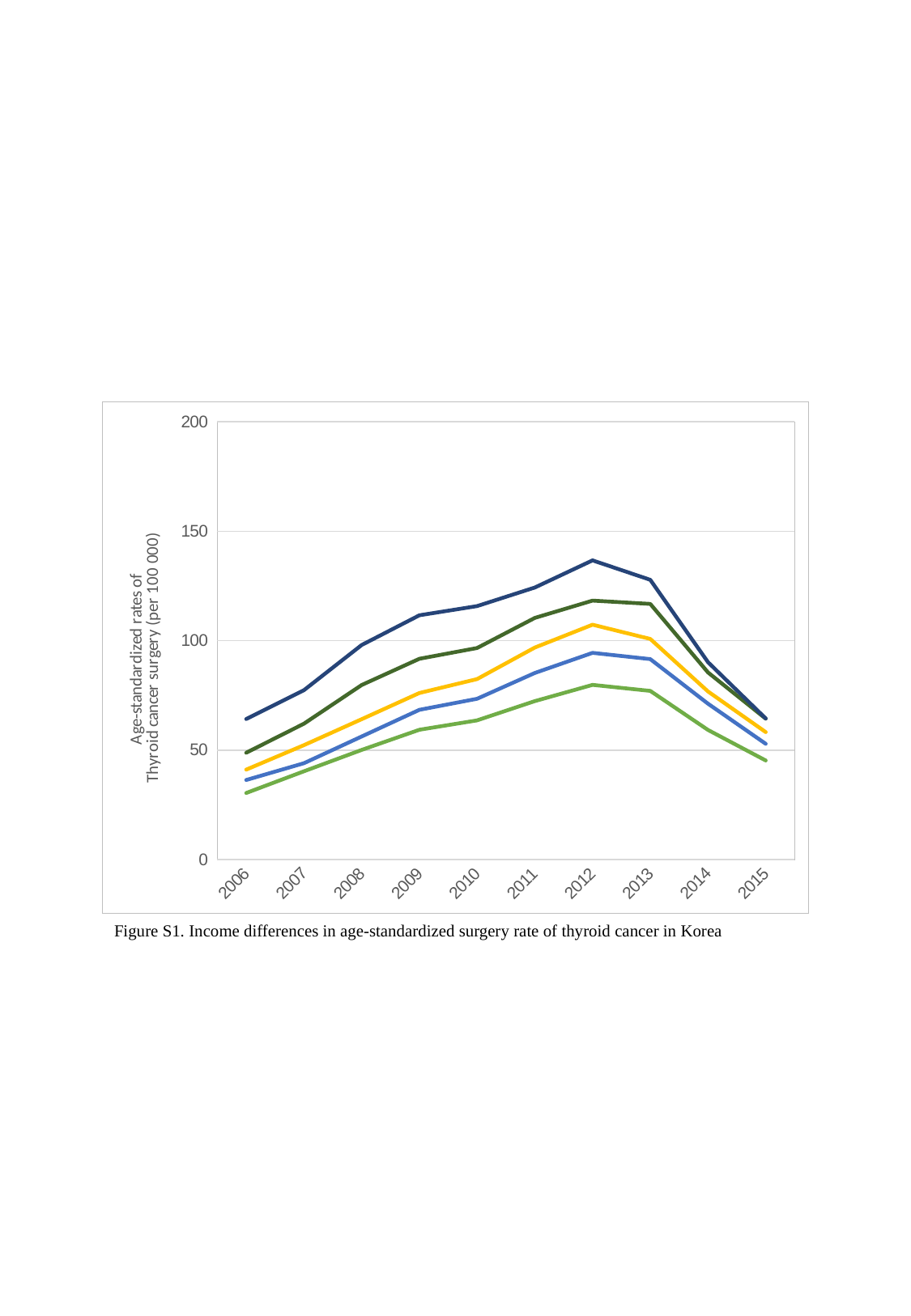

### Chart
| Category | Q1 (lowest) | Q2 | Q3 | Q4 | Q5 (highest) |
|---|---|---|---|---|---|
| 2006 | 30.4003 | 36.339 | 41.0772 | 48.7879 | 64.2013 |
| 2007 | 40.3059 | 44.0146 | 52.2548 | 62.072 | 77.3713 |
| 2008 | 50.0767 | 56.2215 | 64.1079 | 79.7218 | 98.0186 |
| 2009 | 59.299 | 68.3846 | 76.0868 | 91.7202 | 111.6 |
| 2010 | 63.5597 | 73.431 | 82.4343 | 96.6528 | 115.8 |
| 2011 | 72.3733 | 85.2335 | 96.8638 | 110.4 | 124.3 |
| 2012 | 79.786 | 94.4475 | 107.3 | 118.3 | 136.7 |
| 2013 | 77.0392 | 91.5678 | 100.8 | 116.8 | 127.8 |
| 2014 | 59.1986 | 71.1892 | 76.7892 | 85.4659 | 90.1582 |
| 2015 | 45.2607 | 52.869 | 58.2253 | 64.3462 | 64.4442 |Figure S1. Income differences in age-standardized surgery rate of thyroid cancer in Korea
